# How to control the spatiotemporal spread of Omicron in the region with low vaccination rates

**DOI:** 10.3389/fpubh.2022.959076

**Published:** 2022-12-22

**Authors:** Chengzhuo Tong, Zhicheng Shi, Wenzhong Shi, Anshu Zhang

**Affiliations:** ^1^The Department of Land Surveying and Geo-Informatics and Otto Poon Charitable Foundation Smart Cities Research Institute, The Hong Kong Polytechnic University, Kowloon, Hong Kong SAR, China; ^2^School of Architecture and Urban Planning, Research Institute for Smart Cities, Shenzhen University, Shenzhen, China

**Keywords:** Omicron, COVID-19 symptom onset risk, spatiotemporal prediction, vaccination rate, precise control

## Abstract

Currently, finding ways to effectively control the spread of Omicron in regions with low vaccination rates is an urgent issue. In this study, we use a district-level model for predicting the COVID-19 symptom onset risk to explore and control the whole process of spread of Omicron in South Africa at a finer spatial scale. We found that in the early stage of the accelerated spread, Omicron spreads rapidly from the districts at the center of human mobility to other important districts of the human mobility network and its peripheral districts. In the subsequent diffusion–contraction stage, Omicron rapidly spreads to districts with low human mobility and then mainly contracts to districts with the highest human mobility. We found that increasing daily vaccination rates 10 times mainly reduced the symptom onset risk in remote areas with low human mobility. Implementing Alert Level 5 in the three districts at the epicenter, and Alert Level 1 in the remaining 49 districts, the spatial spread related to human mobility was effectively restricted, and the daily onset risk in districts with high human mobility also decreased by 20–80%.

## Introduction

So far, the COVID-19 pandemic is likely to have claimed more than 18 million lives and “battered” the global economy (1). The Omicron variant, which began to spread from South Africa to the rest of the world at the end of 2021, has slowed down an overall global victory over the epidemic (2). In particular, after March 2022, the number of new infections throughout the world increased for the first time since the end of January (3). The largest increase was observed in the Western Pacific Region, with a 25% increase in cases and a 27% increase in death. In Europe, new cases increased by 12%, deaths by 14%, and cases by 2% (3). However, some countries with very high vaccination rates, such as Singapore and the United Kingdom, began to lift restrictions and adapt to a form of coexistence with Omicron (4, 5). This situation did not necessarily apply to low-income/low-middle-income countries and certain high-middle-income countries. The average vaccination rate in 27 low-income countries is currently just over 11%, and that in 55 low- to middle-income countries is < 50% (6). Omicron is observed to be have a lethal impact on regions with low vaccination rates owing to the high infectivity of the disease (7). The mortality rate in low- and low-middle-income countries has been estimated to be 31% higher than that in high-income countries (7). Hence, it is crucial for low-income/low-middle-income countries to prevent and control the epidemic as precisely as possible, by effectively tracking the spatiotemporal spread of Omicron.

Although our previous study on province-level onset risk prediction in South Africa revealed the trend of early spread of Omicron and ways to effectively control its early spread at the province level (8), it could not to meet the current needs of precise epidemic control and economic recovery (9–11). Being at the third year of the COVID-19 pandemic, ensuring normal social and economic activities to a greater extent is one of the most critical factors to be considered in epidemic control, especially for low-income areas with limited vaccination rates (12–15). Therefore, it is critical to explore methods to precisely track and control Omicron at a more refined spatial scale (16–19). Moreover, on the basis of continuously improving vaccination rates in low-income areas, combination of prevention and control measures to better prevent and control the epidemic is also worth exploring.

Considering the need for further exploration, based on the district-level weighted kernel density estimation (WKDE) model (20–23), the prediction of the spatiotemporal COVID-19 symptom onset risk at the finer spatial scale is proposed in this study. In this research, we explored the spatiotemporal spread of Omicron in 52 districts (metropolitan or district municipalities) of South Africa during the first 90 days of the epidemic (i.e., the whole process from the outbreak to the slowdown of the epidemic). Similar to the extended WKDE model applied in nine provinces of South Africa, i) the daily human mobility (24), ii) time-varying vaccination rate (25), vaccination efficiency, iii) daily COVID-19 reproductive number R (26), iv) respiratory pathogen surveillance (27), v) SARS-CoV-2 levels in wastewater (28), vi) COVID-19 cases admitted to sentinel hospital surveillance sites (29), vii) rates of positive COVID-19 tests (30), and viii) social distancing level index (9) in the 52 districts of South Africa were incorporated into the district-level WKDE model. In the previous province-level WKDE model, only full vaccination rates were considered. In the new district-level WKDE model in this study, booster vaccination rates of the Johnson & Johnson COVID-19 vaccine starting from 15 November 2021 in South Africa were incorporated (31). Moreover, due to the lack of data on the vaccination efficiency for Omicron as of December 2021, the vaccine effectiveness data used in the previous province-level WKDE model are based on the effectiveness of the Pfizer-BioNTech vaccine and the Johnson & Johnson vaccine against previous variants. Thus, in the district-level WKDE model, the vaccination efficiency data (32–34) on the Pfizer-BioNTech vaccine and Janssen vaccine against the infection of Omicron are used in this study to improve the performance of the prediction model.

Furthermore, to examine the use of the enhanced WKDE model at the district level, this study explores the control of the spatiotemporal spread of Omicron in the whole process from the outbreak to the slowdown of the epidemic, by increasing vaccination rates and precise and differentiated epidemic alert levels. Unlike previous studies conducted at the provincial level in South Africa that focused on the strengthening effect of vaccination on strict and differentiated outbreak control measures, this study explores the strengthening effect of precise and differentiated epidemic prevention and control measures on increasing vaccination rates. The spatiotemporal data of COVID-19 cases in 52 districts of South Africa from 1 October 2021 to 31 December 2021 are used in this study (35).

## Methods

### Data source

**Figure 1:**
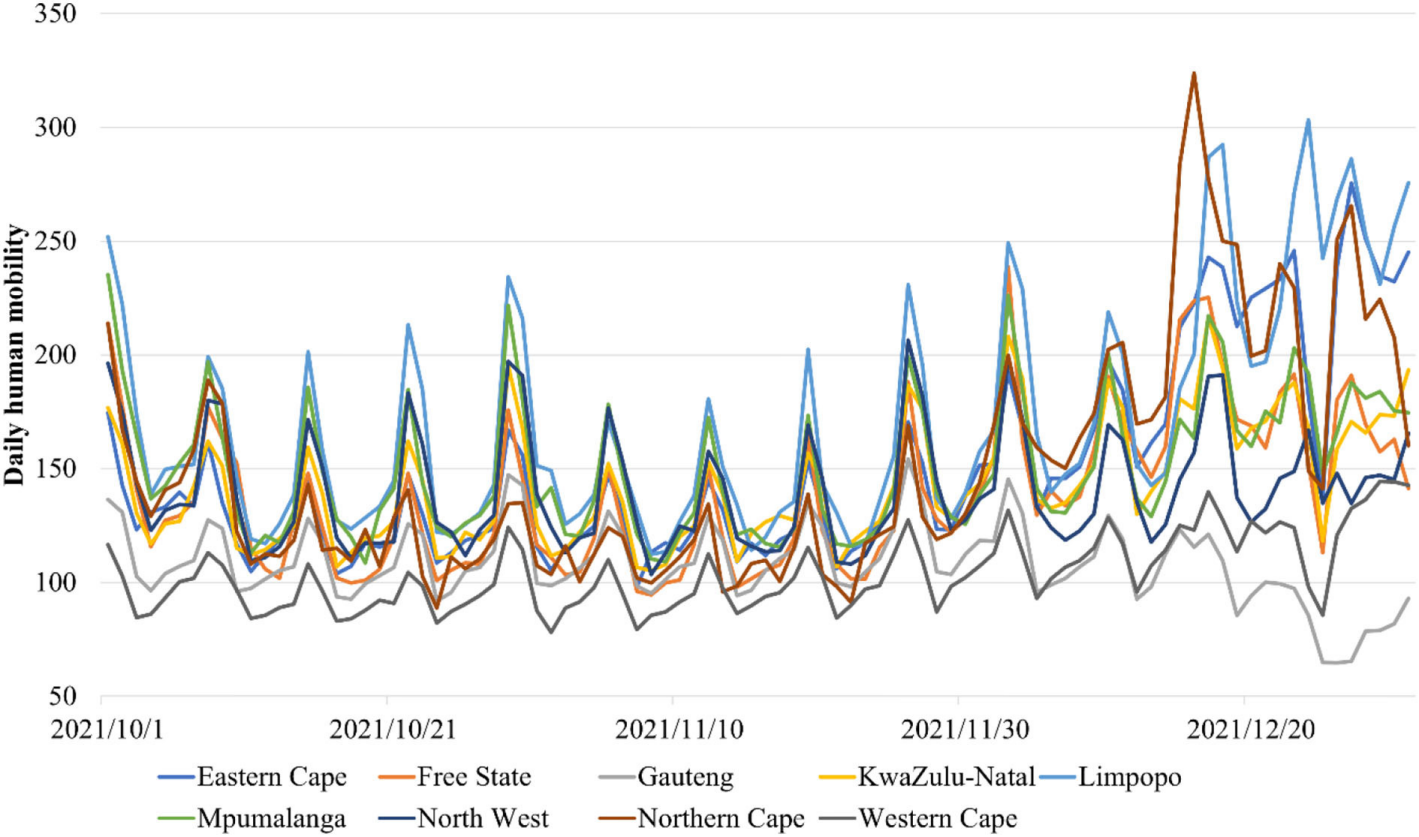
Daily variation of the human mobility in the nine provinces of South Africa from 1 October 2021 to 1 January 2022.

**Figure 2:**
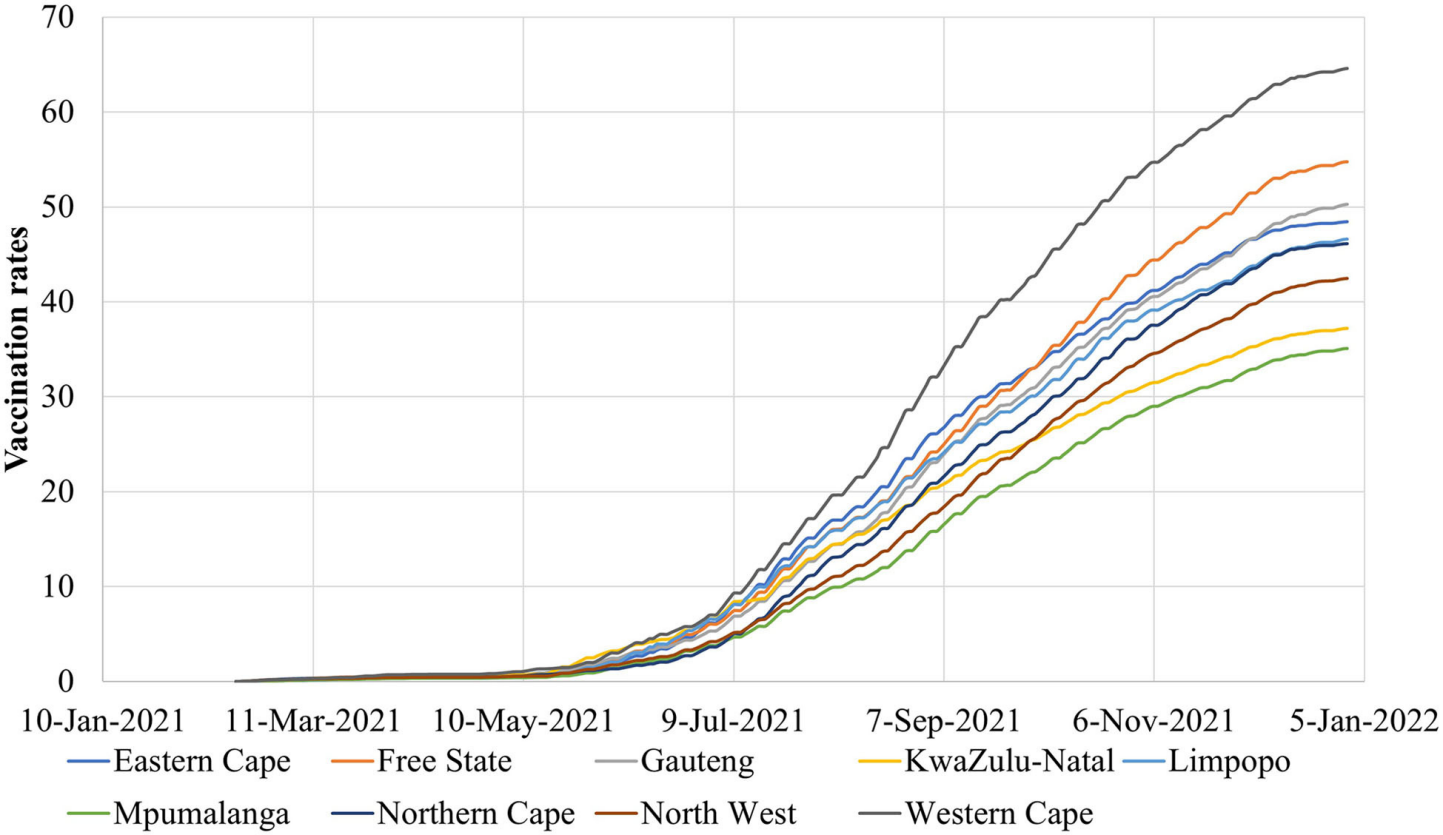
The COVID-19 vaccination rates in the nine provinces of South Africa from 17 February 2021 to 1 January 2022.

Spatiotemporal data of 441,107 COVID-19 cases in 52 districts (metropolitan or district municipalities) from 1 October 2021 to 31 December 2021 were obtained from formal records of the National Institute for Communicable Diseases in South Africa (35). Daily mobility data from 1 October 2021 to 31 December 2021 collected from Apple were used to assess the daily human mobility and the social distancing measures during the COVID-19 epidemic in all 52 districts (Figure 1) (24). Vaccination rates of Johnson & Johnson and Pfizer vaccines in all of the 52 districts of South Africa from 17 February 2021 to 31 December 2021 inclusive were also used (Figure 2) (25). Furthermore, i) daily COVID-19 reproductive number R, ii) the respiratory pathogen surveillance, iii) the rates of positive COVID-19 tests, iv) SARS-CoV-2 in wastewater, and v) COVID-19 cases in sentinel hospital surveillance sites from 1 October 2021 to 31 December 2021 were used in this study.

### A district-level WKDE model for predicting COVID-19 symptom onset risk

As a further development of the original extended WKDE model (8, 20–23), the district-level WKDE model, proposed in this study, includes the same three steps from the extended WKDE model (8, 20–23). The main improvement is that i) V_efficiency_(S, t_i_) denotes the vaccine efficiency against the infection of Omicron in the district with the location S on day t_i_ (8) and ii) V_P_(S, t_i_) is the proportion of the population that have been fully vaccinated and booster vaccinated in the district with the location S on day t_i_ (8).

Moreover, in the new scenario of the Alert Level 5 (i.e., drastic measures to contain the spread of Omicron) in Tshwane, Johannesburg, and Ekurhuleni; the Alert Level 1 (i.e., most normal activity can resume) in the remaining 49 districts in South Africa; and 10 times the current vaccination rates, the daily human mobility *M*(*S, t*_*i*_), and social distancing level factor *M*_*SD*_(*S, t*_*i*_) in Tshwane, Johannesburg, and Ekurhuleni was set as 0. The daily human mobility *M*(*S, t*_*i*_) and social distancing index *M*_*SD*_(*S, t*_*i*_) in 49 other districts were set as usual.

## Results

### The spatiotemporal spread **of** Omicron in 52 districts of South Africa during the whole process from the outbreak to the slowdown of the epidemic

**Figure 3:**
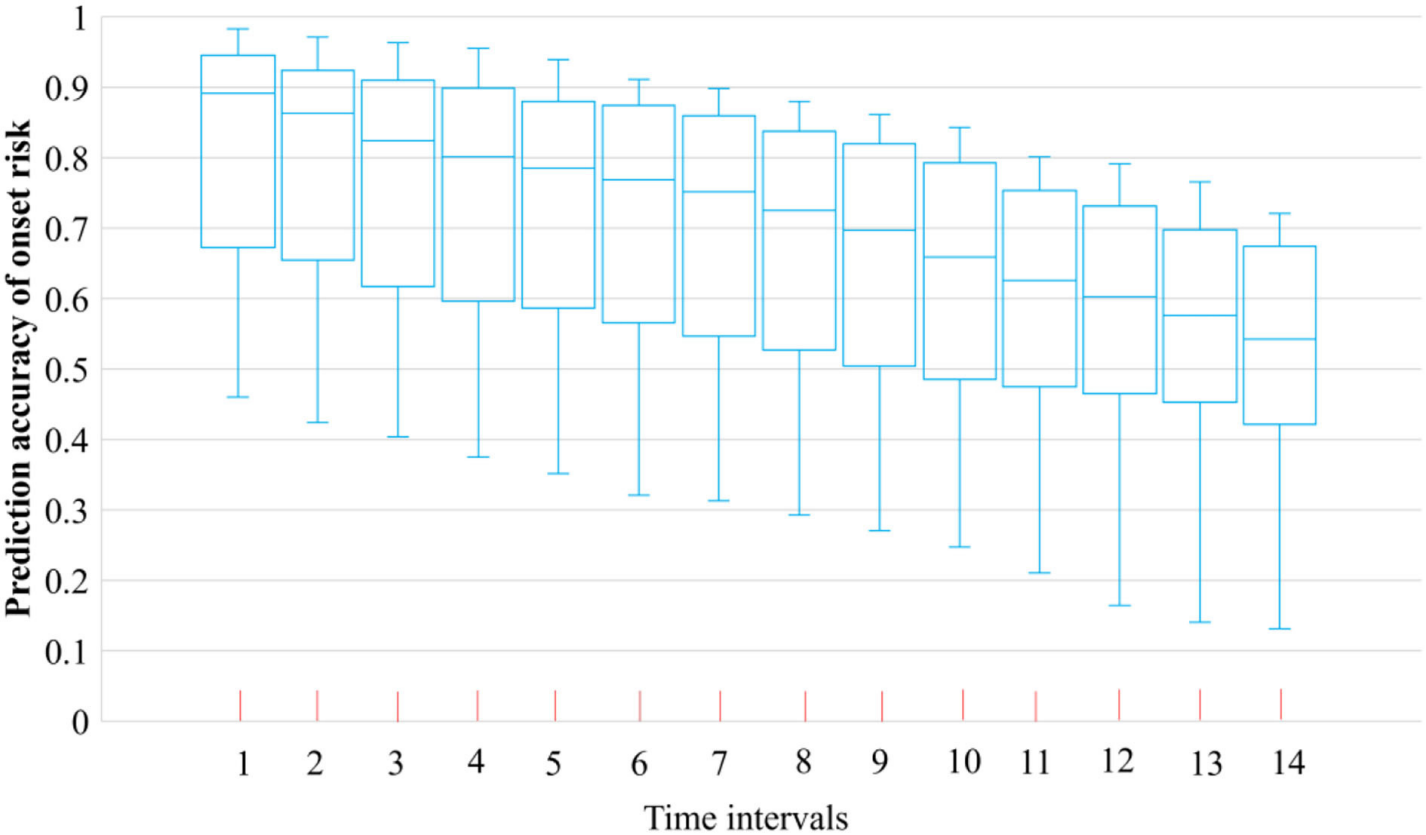
Prediction accuracy of COVID-19 symptom onset risk by the district-level WKDE model.

During the first 90 days of the Omicron spread from 1 October 2021 to 31 December 2021, the COVID-19 symptom onset risk in 52 districts of South Africa was first predicted at the district level by using the new district-level WKDE model. The spatiotemporal information of 441,107 COVID-19 cases in South Africa, during the period from 1 October 2021 to 31 December 2021, was used. The prediction accuracy of the district-level WKDE model was over 75%, with respect to the prediction during the following 7 days (Figure 3).

**Figure 4:**
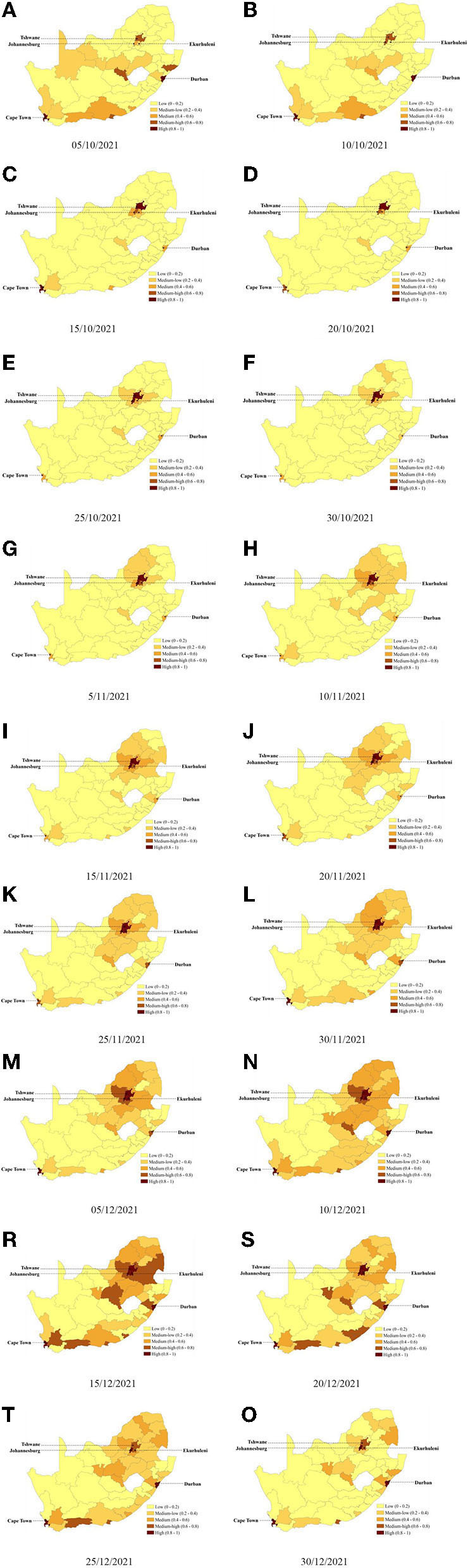
Predicted risk of COVID-19 symptom onset across 52 districts in South Africa **(A–O)** Predicted onset risk during the study period from 5 October 2021 to 1 January 2022. The predicted COVID-19 symptom onset risk was generated using the district-level WKDE model. **(A)** 05/10/2021 **(B)** 10/10/2021 **(C)** 15/10/2021 **(D)** 20/10/2021 **(E)** 25/10/2021 **(F)** 30/10/2021 **(G)** 5/11/2021 **(H)** 10/11/2021 **(I)** 15/11/2021 **(J)** 20/11/2021 **(K)** 25/11/2021 **(L)** 30/11/2021 **(M)** 05/12/2021 **(N)** 10/12/2021 **(R)** 15/12/2021 **(S)** 20/12/2021 **(T)** 25/12/2021, and **(O)** 30/12/2021.

**Figure 5:**
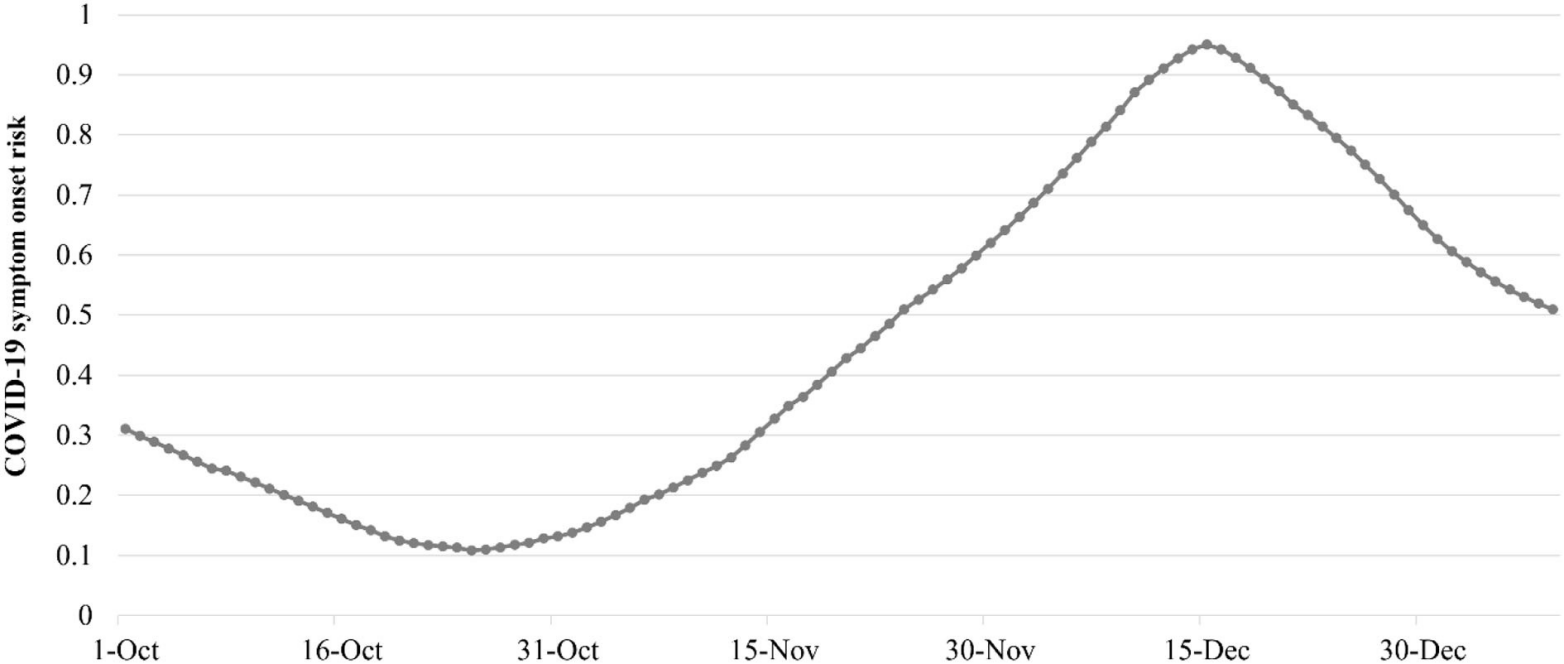
Daily overall risk of COVID-19 symptom onset in South Africa from 1 October 2021 to 1 January 2022.

The first 90 days of the spread of Omicron are analyzed by the spatiotemporal variation of the COVID-19 symptom onset risk as follows (Figure 4): From the beginning of October 2021, it is seen that the onset risk values and levels of 50 districts outside Tshwane and Johannesburg had mostly decreased. For example, the cities of Cape Town and Durban, which were previously at the high onset risk level, had been reduced and termed cities within the medium or medium-low onset risk category (Figure 4F). However, the onset risk value and level in Tshwane continued to increase. By mid-October, this district became the only district termed as “high onset risk.” During this phase, the onset risk in neighboring Johannesburg also increased. By the end of October, Johannesburg was also termed a high-onset risk district (Figure 4F). A previous official analysis of Omicron genomes showed that the Omicron virus had, in fact, possibly begun to appear in Tshwane in early October, before spreading to the neighboring Johannesburg, occasioned by the close transport links. In the initial stage of Omicron emergence, high-risk areas were basically maintained in the two districts of Tshwane and Johannesburg in the Greater Johannesburg area. This suggests that during this early stage, the spatiotemporal trend of Omicron was relatively slow. According to South Africa's official report, the earliest detection of Omicron in South Africa was in Tshwane, thus supporting the results of our analysis of the incidence of Omicron. However, throughout October, the onset risk in the other 50 regions mostly showed a downward trend. Therefore, the overall symptom onset risk in South Africa decreased from 0.31 to 0.13 (Figure 5), indicating that the epidemic outbreak, caused by the dominant Delta variant in October 2021, was gradually waning and being replaced by the more transmissible Omicron.

The Omicron variant became the dominant variant in early November 2021, and the intensity of the spatial spread gradually increased, especially in districts close to Tshwane and Johannesburg, the epicenter of the outbreak. A total of 20 northern districts around Tshwane and Johannesburg, near the epicenter of the outbreak, all became the regions with increased onset risk (Figures 4G–I). There were 11 districts with increased onset risk to a medium-low onset risk level and six regions with increased onset risk increased to a medium onset risk level. Ekurhuleni, which borders both Tshwane and Johannesburg, was the most severely affected and gradually became the medium-high-onset risk and high-onset risk area in early and late November 2021, respectively (Figure 4L). In addition, Omicron has also spread from Tshwane and Johannesburg, the epicenter of the outbreak, to Cape Town and Durban, where traffic was well connected. Cape Town and Durban are the second and third largest cities in South Africa, which are important nodes in South Africa's domestic and external transportation network, as well as tourism and economic hot spots. In both places, the onset risk had risen from the original medium level to above the medium-high level. The onset risk level in their neighboring cities also increased from low onset risk to medium-low onset risk. Correspondingly, the overall onset risk in South Africa on 31 November 2021 increased by 3.7 times, reaching 0.62 (Figure 5). In the first half of December 2021, Omicron continued to spread to the surrounding districts affected by heavy traffic to Johannesburg (Figures 4M–R). At the same time, onset risk was increasing in districts adjacent to both Tshwane and Johannesburg. By 15 December 2021, the epidemic outbreak caused by Omicron had reached its peak. The onset risk in 13 districts was at a medium-high onset risk level, and the onset risk in 10 districts was at a medium onset risk level. The overall onset risk in South Africa also increased from 0.62 to 0.95 during this period (Figure 5).

The epidemic peak was reached on 15 December 2021, during the following 15 days; apart from the onset risk in Johannesburg, Cape Town, and Durban, which remained at a high onset risk level, the onset risk in other districts decreased, especially in districts around traffic hot spots (Figures 4R–O). The onset risk levels in 20 districts were reduced from the original medium-high, medium, or medium-low onset risk level to a low risk level. Even in Tshwane, the previous epicenter of the outbreak dropped to a medium-high onset risk level. During this period, the overall onset risk in South Africa decreased by 34.04% (Figure 5). After January 2022, the overall onset risk in South Africa continued to drop to 0.51, which is a medium onset risk level (Figure 5).

### To control the spatiotemporal spread of Omicron in 52 districts of South Africa during the whole process from the outbreak to the slowdown of the epidemic

Despite a 2.96–5.45% increase in vaccination coverage across South Africa during the months following 28 November 2021, the overall symptom onset risk still increased by 64.45%. A limited increase in vaccination rates had not been effective in containing the outbreak. Hence, explored in this study is the control effect for Omicron when the vaccination rates of 52 districts in South Africa could be increased to above 90%. Thus, based on the predicted risk of the COVID-19 symptom onset in the 52 districts of South Africa, this control effect for Omicron was analyzed and compared by the simulation conducted in i) the current daily vaccination rates in 52 districts and ii) 10 times the current daily vaccination rates in 52 districts.

**Figure 6:**
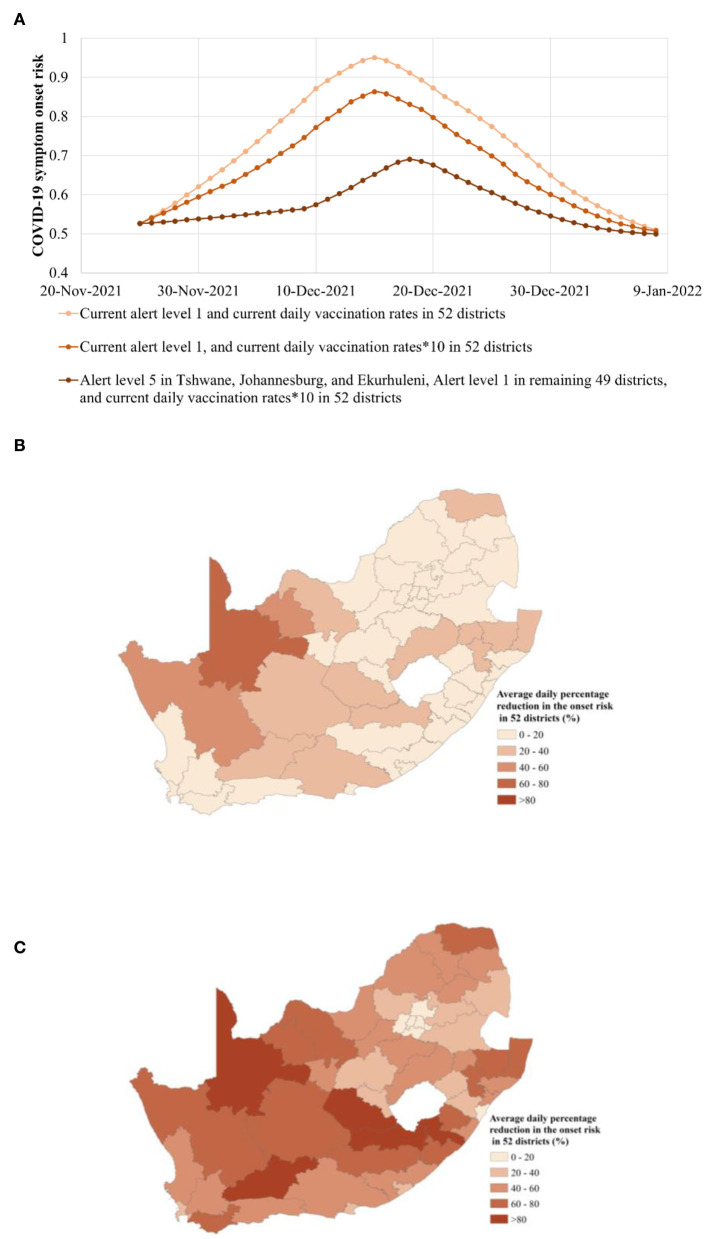
Risk of COVID-19 symptom onset in the three epidemic alert scenarios of i) Alert Level 1 in all 52 districts, ii) Alert Level 1 in all 52 districts, and 10 times vaccination rates, and iii) Alert Level 5 in Tshwane, Johannesburg, and Ekurhuleni together with Alert Level 1 in the remaining 49 districts, and 10 times vaccination rates from 26 November 2021 to 5 January 2022. **(A)** Overall onset risk in the three epidemic alert scenarios. The plotted values were computed with the predicted risk of COVID-19 symptom onset in the four epidemic alert scenarios. **(B)** Average daily percentage reduction in the onset risk in 52 districts, regarding the scenario of the Alert Level 1 in all 52 districts, and 10 times vaccination rates in all 52 districts, when compared with the scenario of the current Alert Level 1 and the current vaccination rates for the entire South Africa region. **(C)** The average daily percentage reduction in the onset risk in 52 districts, regarding the scenario of the Alert Level 5 in Tshwane, Johannesburg, and Ekurhuleni together with Alert Level 1 in the remaining 49 districts, and 10 times vaccination rates, when compared with the scenario of the current Alert Level 1 and the current vaccination rates for the entire South Africa region.

The comparison between the daily overall onset risk values in South Africa and the current vaccination rates showed that the rates reflect the precise effect of the improved vaccination rates. A constant lower daily overall onset risk was found after increasing the current vaccination rates to 10 times (Figure 6A). It was then found that when the daily vaccination rate in each district was increased by 10 times, the daily overall onset risk in South Africa was only reduced by 0.41%−11.38% (Figure 6A). Although the overall onset risk of South Africa had decreased, it still remained at a high onset risk level of 0.86 (Figure 6A). Overall, even a 10-fold increase in daily vaccination rates appears to have a limited effect on curbing the spread of Omicron in countries such as South Africa, in which vaccination rates are low with an intermediate risk level prior to the increase in the vaccination rates. Furthermore, the onset risk in all the 52 districts, which had 10 times the current daily vaccination rates, on the same date, was obviously lower than that with the current daily vaccination rates, especially for the districts far from the epicenter of the epidemic and the important nodes of transportation (Figure 6B). For example, John Taolo Gaetsewe, Namakwa, and ZF Mgcawu, located in the northwest of South Africa, had a 41% to 65% lower risk. However, the number of districts with a risk reduction of < 20% was 37, reaching to 71.15% (Table 1). Most of these districts are in the vicinity of the epidemic center and important transportation nodes. This appears to suggest that increasing the initial vaccination rate has a limited effect on reducing the spatial spread of the Omicron status, enhanced by the human mobility.

**Table 1:** Risk reduction of COVID-19 symptom onset in different scenarios of (i) Alert Level 1 in all 52 districts, and 10 times vaccination rates, and (ii) Alert Level 5 in Tshwane, Johannesburg, and Ekurhuleni together with Alert Level 1 in the remaining 49 districts, and 10 times vaccination rates from 26 November 2021 to 5 January 2022.

**Risk reduction (%)**	**Alert level 1 in all 52 districts, and 10 times vaccination rates**	**Alert Level 5 in Tshwane, Johannesburg and Ekurhuleni together with Alert Level 1 in the remaining 49 districts, and 10 times vaccination rates**
0–20	37	6
20–40	12	11
40–60	2	18
60–80	1	12
80–100	0	5

How to reduce the spatial spread of Omicron due to enhanced population movement was further explored. This study has explored the effect of strict control measures adopted in limited high-onset risk districts at the epicenter of the outbreak, while general measures have been adopted in other districts. For more than a month prior to 26 November 2021, the high-risk areas were present largely at Tshwane, the source of the outbreak, and surrounding Johannesburg and Ekurhuleni. Thus, on the basis of 10 times the vaccination rate, the simulation was conducted from 26 November 2021 in the new scenarios of Alert Level 5 in Tshwane, Johannesburg, and Ekurhuleni, together with Alert Level 1 for the remaining 49 districts. On the basis of the 10 times vaccination rates in 52 districts, compared with the current scenario of Alert Level 1, the daily overall onset risk in the new scenario has further declined by 1.58–26.14% and has been maintained below the medium-high risk level (Figure 6A). Approximately 14 days after Alert Level 5 in Tshwane, Johannesburg and Ekurhuleni, together with Alert Level 1 in the remaining 49 districts were implemented, the rising speed of daily overall onset risk was 82.29% lower, on average, than the Alert Level 1 scene (Figure 6A). Compared with the scenario of Alert Level 1 and 10 times the vaccination rates, the peak of the overall onset risk was also delayed by 3 days (Figure 6A). However, compared with the current scenario of Alert Level 1 and 10 times the vaccination rates, in the stage of epidemic mitigation, the decline trend of onset risk in the new scenario was correspondingly slowed down. This may be related to the fact that symptomatic infection under the new measures was greatly reduced, and the immune barrier could not be formed sufficiently quickly.

Furthermore, in the new scenario of Alert Level 5 in Tshwane, Johannesburg, and Ekurhuleni, together with Alert Level 1 in the remaining 49 districts, the spatial differences in the reduction of onset risk in various provinces have also been further explored (Figure 6C). It can be seen that compared with the Alert Level 1 in 52 districts, the onset risk in remote districts far from the epicenter of the epidemic outbreak and transportation hot spots has further dropped significantly. The onset risk in five of these districts even dropped by more than 80% (Figure 6C, Table 1). Furthermore, the daily onset risk in i) the neighboring districts of the epicenter and ii) districts with high human mobility also decreased by 20–80%. Of particular concern was the average drop in onset risk of over 21% in Cape Town, the second largest city, and Durban, the third largest city. In addition, the Western Cape and KwaZulu-Natal, the economically important provinces in which Cape Town and Durban are located, experienced an average reduction of 51% and 48% in the onset risk (Figure 6C). This not only has a positive effect regarding controlling the spread of the epidemic along the transportation flow network but also helps maintain normal socioeconomic activities in such key tourism and manufacture-oriented cities. The slight Alert Level 1 adopted by 49 districts outside the three high-onset risk districts did not show a huge impact on the economic and social life of most districts, as had the global Alert Level 4 adopted by South Africa in controlling the Delta variant.

## Discussion

A few regions of the world are currently relying on high vaccination rates to effectively prevent the spread of the highly contagious Omicron variant of SARS-CoV-2. However, people in these low- and low-middle-income regions with low vaccination rates are experiencing a considerable outburst. Although the death rate caused by Omicron was somewhat low, it was not the same among unvaccinated people. According to the WHO COVID-19 Strategic Preparedness and Response Plan for 2022 (36), control measures must continue to be explored and continued to better protect susceptible populations based on better monitoring and Omicron tracking and faster and more accurate identification of to where it spreads.

On the basis of previous research on the tracking of Omicron at the provincial scale in South Africa in the early spread stage, at the finer spatial scale, this study explored the spatiotemporal dynamics of the Omicron spread in the 52 districts of South Africa during the whole process from the outbreak to the slowdown of the epidemic. The results are as follows:

i) The spatiotemporal spread of Omicron during the first 30 days was mainly concentrated in the surrounding areas such as Tshwane and Johannesburg, the source of the outbreak. The overall risk of COVID-19 dropped by 58% to 0.13 as outbreaks caused by the Delta epidemic in other regions were gradually receding.
ii) After becoming the dominant variant in South Africa, Omicron has continued to spread from Tshwane and Johannesburg, the epicenter of the outbreak, to neighboring regions and also along the population flow network to other traffic hot spots such as Cape Town and Durban. The spread of Omicron further expanded to areas around traffic hot spot districts. Although the South African government had stepped up its call for vaccination during this period, there appears to be limited reduction in the onset risk caused by Omicron. When the Omicron outbreak in South Africa peaked, the overall risk increased 5.59-fold at high onset risk levels. Among them, the onset risk in key districts of economic development in traffic hot spots also increased to the medium-high onset risk level, a feature not conducive to the overall economic development of South Africa.
iii) After the peak of the outbreak on 15 December 2021, except for Johannesburg, Cape Town, and Durban, the disease decreased rapidly, especially in areas around traffic hot spots. The spatiotemporal spread of Omicron thus appeared to rapidly weaken along the transportation network. During this period, however, the overall risk of the disease, further developing in South Africa, dropped to 0.51, which is the medium onset risk level.

Together with increasing the vaccination rate, ways to prevent the spatiotemporal spread of Omicron by the adoption of precise and differentiated control measures, rather than severe restrictions on the whole region, have been further explored in the following three scenarios: i) the actual Alert Level 1 and the daily vaccination rates in 52 districts, ii) the actual Alert Level 1 and 10 times the daily vaccination rates in 52 districts, and iii) the Alert level 5 in Tshwane, Johannesburg, and Ekurhuleni together with Alert Level 1 in the remaining 49 districts, and 10 times the daily vaccination rates in 52 districts. The results regarding recorded attempts to control the spatiotemporal spread of Omicron in 52 districts of South Africa are as follows:

i) Even a 10-fold increase in daily vaccination rates appears to have a limited effect on containing the spread of Omicron in regions such as South Africa, in which vaccination rates were low and with an intermediate risk early in the outbreak. The overall daily risk in South Africa was only reduced by 0.41–11.38% and remained at a high onset risk level of 0.86. This containment effect was practiced mainly to reduce risk in remote areas far from the epidemic center and important transportation nodes. The risk was reduced by < 20 % in most traffic hot spots, suggesting that a timely and substantial increase in vaccination rates has a limited impact on reducing the spatial spread of Omicron by means of human mobility.
ii) On the basis of the “10 times vaccination rates,” under the new scenario of Alert Level 5 in the three districts of Tshwane, Johannesburg, and Ekurhuleni at the epicenter of the outbreak, and Alert Level 1 in the remaining 49 districts, the daily overall onset risk was further reduced by 1.58–26.14% and remained below the medium-high risk level (Figure 4A). In the early stage of the Omicron spread, the rate of increase in the daily overall onset risk was reduced by 82.29% compared with that in the actual Alert Level 1 scenario. The peak overall onset risk was also delayed by 3 days. In the mitigation stage of the epidemic, the decreasing trend of the overall onset risk slowed down due to the inability to form an immune barrier through a large number of infections. However, this scenario played a significant role in reducing the spread *via* the population movement. Notably, the daily onset risk in districts with high human mobility also decreased by 20–80%. For example, Western Cape and KwaZulu-Natal, economically important provinces, had an average of 51% and 48% lower onset risk, respectively. This not only had a positive effect on controlling the spread of the epidemic along the traffic flow network but also helped the aforementioned key tourism and manufacturing cities to maintain the practice of normal socioeconomic activities. Moreover, compared with the Alert Level 5 in all districts of Gauteng Province in the previous study, the Alert Level 5 measures adopted in only three districts in this study showed a reduction in the daily overall onset risk by 1.58–26.14% in the whole process of Omicron spread. The risk reduction effect of the measures taken in this study appears to be more effective. This also avoids a negative impact on the normal socioeconomic activities of an additional 3.06 million people.

A total of three million deaths due to COVID-19 occurred during the 3 months following the emergence of the Omicron variant (7), especially in low-income regions. While most cases emerging were found to be mild, their sheer number obviously suggested the death toll would remain high (7). As the current vaccination coverage in low-income areas is still too low to establish an immune barrier, precise and differentiated epidemic prevention measures are required to complement the ever-increasing vaccination to protect more people. Compared with the previous study on the early spread progress of Omicron conducted in nine provinces of South Africa, this study has comprehensively investigated the whole process of spatiotemporal diffusion of Omicron on a finer district scale, especially for the process of gradual weakening of the spread of Omicron in the late stage. More importantly, by predicting and simulating at the finer spatial scale, the implementation of stringent higher order control measures can be carried out in a smaller area, while most normal activities in other larger areas can be maintained. These results have shown that the epidemic control effect of this finer scale prevention and control is not weaker than that of the previous large-scale high-level prevention and control at the provincial level. This is critical for low-income areas that desperately need restoration of their economies and secure livelihoods.

Thus, details revealed in the article could assist countries and regions with low vaccination rates to further control the spatiotemporal spread of Omicron through more precise and differentiated control measures by continuously increasing vaccination rates, thereby reducing the potential negative social health impacts and better enabling the continuation of normal economic and social activities. Therefore, it is expected that this study could help countries and regions better implement precise epidemic prevention measures to defeat the epidemic at an early stage and thus enabling the continuation of normal economic and social development.

## Data availability statement

The original contributions presented in the study are included in the article/supplementary material, further inquiries can be directed to the corresponding author.

## Author contributions

CT collected the data, developed the computation models, wrote the manuscript, and interpreted the results. ZS wrote the manuscript, collected the data, analyzed the data, and interpreted the results. WS conceived, designed the study, interpreted the results, developed the computation models, analyzed the data, and wrote the manuscript. AZ developed the computation models, analyzed the data, and wrote the manuscript. All authors contributed to the article and approved the submitted version.
